# Retraction: Monocrotophos Induces the Expression and Activity of Xenobiotic Metabolizing Enzymes in Pre-Sensitized Cultured Human Brain Cells

**DOI:** 10.1371/journal.pone.0214164

**Published:** 2019-03-15

**Authors:** The PLOS ONE Editors

Following publication of this article [1], concerns were raised regarding the following figure panels:

- Figure 4A: Similarity between all bands in 3-MC β-actin panel
- Figure 4A: Similarity between all bands in 3-MC+MCP β-actin panel
- Figure 4A and C: Similarity between β-actin panel of MCP treated SH-SY5Y cells
- Figure 4B and D: Similarity between β-actin panel of MCP treated U373-MG cells
- Figure 4C: Similarity between lanes 2, 3 and 4, 5 in the β-actin panel of CPA treated SH-SY5Y cells
- Figure 4D: Similarity between lanes 2, 3, 4 of the β-actin panel of CPA treated U373-MG cells and lanes 1, 2, 3 of the β-actin panel of CPA + MCP treated U373-MG cells
- Figure 5A: Similarity between 72 h and 96 h images for CYP1A1 in SH-SY5Y cells 3MC group
- Figure 5B: Similarity between 3-MC treated 72 h microscopy image and 3-MC+MCP 24 h image
- Figure 5B: Similarity between 3-MC + MCP treated 48 h and 72 h images
- Figure 5B: Similarity between 24 h 3-MC and 96 h 3-MC images
- Figure 7B: Similarity between the images for 96 h ethanol treated and 96 h ethanol+ MCP treated CYP2E1 in U373-MG cells

The authors explained that the duplications in microscopy images were the result of errors during figure preparation and have provided replacement images to address the microscopy image duplications.

For Figure 4C β-actin panel of CPA treated SH-SY5Y cells, Figure 4D β-actin panel of CPA treated U373-MG cells and Figure 4D β-actin panel of CPA + MCP treated U373-MG cells, the authors have provided replacement images that they claim represent replication experiments carried out at the same time.

In the case of Figure 4A, the authors stand by the validity of the 3-MC β-actin panel and 3-MC+MCP β-actin panel.

The authors have explained that the β-actin panel of MCP treated SH-SY5Y cells in Figure 4A and 4C is the same; and the β-actin panel of MCP treated U373-MG cells in Figure 4B and 4D is the same. These panels represent the same experimental samples and conditions.

However, the raw data supporting the published blots, including those for which concerns have not been raised, are unavailable.

In light of the outstanding concerns that cast doubt on the integrity of the published findings, the *PLOS ONE* Editors retract this article.

VT, VK, MK, SJ, SA, SY did not respond. AKS, AP, FK, VKK, ML, ABP did not agree with the retraction.
